# Treatment Outcomes for Primary Hepatic Angiosarcoma: National Cancer Database Analysis 2004–2014

**DOI:** 10.3390/curroncol29050292

**Published:** 2022-05-17

**Authors:** Ankit Mangla, Gino Cioffi, Jill S. Barnholtz-Sloan, Richard T. Lee

**Affiliations:** 1 Seidman Cancer Center, University Hospitals, Cleveland, OH 44106, USA; rtl24@case.edu; 2 Department of Population and Quantitative Health Sciences, Case Western Reserve University School of Medicine, Cleveland, OH 44106, USA; gino.cioffi@nih.gov (G.C.); jill.barnholtz-sloan@nih.gov (J.S.B.-S.)

**Keywords:** angiosarcoma, database, surgery, chemotherapy, academic medical center

## Abstract

**Simple Summary:**

Primary hepatic angiosarcoma is a rare tumor of the liver. The prognosis and treatment of this rare tumor remains an enigma. Despite being the most common liver tumor of mesenchymal origin, the prognosis of patients diagnosed with primary hepatic angiosarcoma has never been compared with that of the most common liver tumor which is hepatocellular carcinoma. In this manuscript, we have analyzed all the recorded cases in the National Cancer Center Database and determine the best approach to treat this rare tumor. We have also conducted a brief review of the literature to guide the reader toward the finer nuances of managing patients diagnosed with primary hepatic angiosarcoma, especially in the context of a liver transplant.

**Abstract:**

Background: To determine the risk of mortality and factors associated with survival amongst patients diagnosed with primary hepatic angiosarcoma (PHA). Methods: All patients diagnosed with hepatocellular carcinoma (HCC) or PHA from 2004 to 2014 were identified from the National Cancer Database (NCDB). Further analysis was performed within the cohort of patients with PHA to assess the impact of surgery, chemotherapy, radiation, and facility type on overall survival (OS). A multivariable analysis using the Cox proportional methods and a survival analysis using the Kaplan–Meier method were used. Results: A total of 117,633 patients with HCC were identified, out of whom 346 patients had PHA. Patients with PHA had a mean age of 62.9 years (SD 13.7), the majority were men (64.7%), white (85.8%), and had a Charlson comorbidity index (CCI) of zero (66.2%). A third of the patients with PHA (35.7%) received chemotherapy, and 14.6% underwent a surgical resection. The median survival was 1.9 months (1.8–2.4 months) compared to patients with HCC (10.4 months, 10.2–10.5) (aHR-2.41, 95% CI: 2.10–2.77, *p* < 0.0001). Surgical resection was associated with a higher median survival (7.7 versus 1.8 months, aHR-0.23, 95% CI: 0.15–0.37, *p* < 0.0001). A receipt of chemotherapy was associated with a higher median survival than no chemotherapy (5.1 versus 1.2 months, aHR-0.44, 95% CI: 0.32–0.60, *p* < 0.0001), although the survival benefit did not persist long term. Conclusion: PHA is associated with poor outcomes. A surgical resection and chemotherapy are associated with improved survival outcomes; however, the long-term benefits of chemotherapy are limited.

## 1. Introduction

Primary hepatic angiosarcoma (PHA) is an extremely rare malignancy with an estimated incidence of 0.5–2.5 cases per 10 million people [1,2]. Despite being rare, PHA is the most common mesenchymal tumor of the liver [3]. Several chemical compounds such as vinyl chloride, colloidal thorium dioxide, androgenic steroids, and phenylhydrazine are implicated in the development of PHA. Hereditary conditions such as hemochromatosis and neurofibromatosis are also associated with PHA [3,4]. A latency period of 10–40 years is noted between exposure to a carcinogen and the development of PHA [1,3,4]. A clinical diagnosis of PHA requires a high index of suspicion as most patients will present with non-specific symptoms, such as abdominal pain, weight loss, and fatigue. Imaging is needed to differentiate PHA from benign lesions and other malignant tumors. Contrast-enhanced computed tomography (CT) of the liver shows a multifocal, heterogenous, vascular tumor with internal hemorrhage in larger lesions and a disordered patchy arterial phase enhancement which is progressive in later phases [5,6]. Histopathology shows large pleomorphic sinusoidal cells. Immunohistochemistry is positive for endothelial markers (von Willebrand factor, *Ulex europaeus* agglutinin 1, and CD 31 are the most reliable markers amongst several others) [3,7,8].

PHA is an aggressive malignancy and tends to metastasize quickly within the liver or distant organs. Surgical resection is offered to patients with localized disease. In patients with localized PHA, resection of the tumor with an R0 margin (microscopic negative margin) is associated with the best survival outcomes [9,10,11]. Patients diagnosed with metastatic PHA are treated with systemic therapy only. However, neoadjuvant systemic therapy is sometimes offered to patients with localized disease to help increase the chances of achieving an R0 resection. Adjuvant therapy has also been used in patients undergoing surgical resection in an attempt to increase the progression-free survival [11,12]. Because PHA is a rare disease, most data in the literature come from case reports and short case series. We conducted this study utilizing the National Cancer Database (NCDB) to explore the real-world outcomes of patients diagnosed with this extremely rare tumor. Although hepatocellular carcinoma (HCC) and PHA are two different malignancies, they are aggressive and associated with poor outcomes. There has never been a formal comparison of the difference in outcomes of the two cancers. Hence, we also incorporated data from patients with hepatocellular carcinoma (HCC) as a reference to patients with PHA to compare the outcomes of the two malignancies.

## 2. Materials and Methods

Adults (ages 18+) diagnosed with HCC or PHA were identified in the National Cancer Database (NCDB) from 2004 to 2014. International Classification of Diseases for Oncology, Third Edition (ICD-O-3) codes were used for the identification of HCC (8170/3) and PHA (9120/3). Descriptive statistics for clinical and sociodemographic characteristics were obtained. Chi-square test was used to assess differences in categorical variables, and t-tests were performed to assess differences in continuous variables. These tests were also performed within PHA patients to compare characteristics between surgery status and chemotherapy status. The Kaplan–Meier method with log-rank test was used to calculate survival for HCC and PHA patients. Additional analyses were performed on the PHA cohort to assess the impact of surgery and facility type on overall survival. Univariate and multivariable Cox proportional hazards models were performed to assess differences in survival. Multivariable analyses were adjusted for age, sex, Charlson–Deyo comorbidity index (CCI) score, race, and ethnicity, urbanicity, insurance status, facility location and type, treatment received (surgery, chemotherapy, or RT) were performed, and adjusted hazard ratios (aHR) are reported. Statistical significance was set at <0.05. All analyses were performed using SAS version 9.4 (SAS institute, Cary, NC, USA). The study was approved by the institutional review board at Case Western Reserve University. The study was performed in accordance with relevant guidelines and regulations.

## 3. Results

A total of 117,633 patients with HCC and 346 patients with PHA were identified in the database. PHA was diagnosed in patients with a mean age of 62.9 years (SD: 13.7 years) and had male preponderance (male versus female: 64.7% versus 35.3%, respectively). Most patients belonged to the white race (85.8%), and most were treated either in an academic/research program or in a comprehensive community cancer program (49.8% and 31.6%, respectively). Only 14.6% of patients received surgery, 3.5% received RT, and 35.7% received chemotherapy (Table 1). Twelve patients (3.47%) with PHA received chemotherapy and surgery, six patients (1.74%) received chemotherapy and RT, and two patients (0.58%) received surgery and RT. No patient recorded in the database received all three treatment modalities. Among the patients with PHA, those who received surgery had a median survival of 7.7 months (5.5–16.9), compared to 1.8 months (1.5–1.9) for those who did not receive surgery. When adjusted for demographic factors, such as age, sex, race, ethnicity, and location, and clinical factors, such as comorbidities and treatment, patients who received surgery had a 77% decrease in the risk of death compared to those who did not receive surgery (aHR-0.23, 95% CI: 0.15–0.37, *p* < 0.0001). There was a similar trend among those who received chemotherapy, having a median survival of 5.1 months (3.8–5.8) as compared to 1.2 months (0.9–1.6, *p* < 0.0001) without chemotherapy. Adjusting for the same factors as above, those patients who received chemotherapy had a 56% decrease in the risk of death than those who did not (aHR-0.44, 95% CI-0.32–0.60, *p* < 0.0001) (Table 2). Among patients diagnosed with PHA, those who received surgery were significantly younger than those who did not (55.9 years vs. 64.2 years, *p* < 0.001), as well as those who received chemotherapy (59.7 years vs. 65.0 years, *p* < 0.001). (Table 3). Patients treated at an academic center had a higher median survival (2.9 months, 95% CI: 2.2–4.1) than those treated at a non-academic center (1.9 months, 95% CI: 1.2–2.4). However, there was no significant difference in the overall survival of patients treated at an academic center (aHR-0.99, 95% CI: 0.74–1.34), *p* = 0.97).

### Primary Hepatic Angiosarcoma versus Hepatocellular Carcinoma

**Figure 1:**
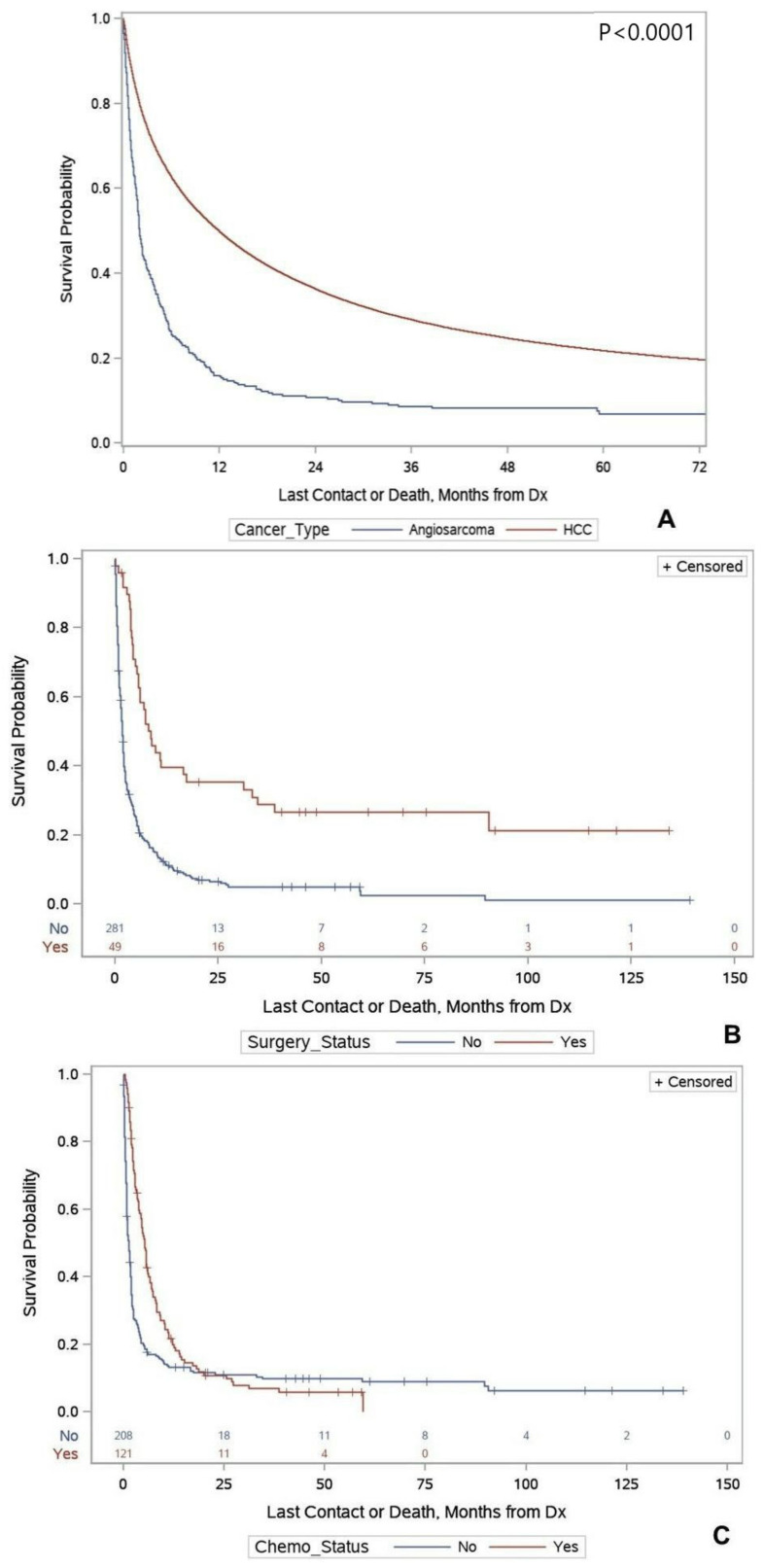
Panel (**A**): Kaplan–Meier Survival curves for patients diagnosed with primary hepatic angiosarcoma and hepatocellular carcinoma. Panel (**B**): Kaplan–Meier Survival curves for surgery status for patients diagnosed with primary hepatic angiosarcoma. Panel (**C**): Kaplan–Meier Survival curves for chemotherapy status for patients diagnosed with primary hepatic angiosarcoma, patients diagnosed with primary hepatic angiosarcoma. Data from National Cancer Database 2004–2014.

A notably larger proportion of female patients with PHA than those with HCC were recorded in the NCDB database (35.3% vs. 23.3%, *p* < 0.0001). A higher proportion of white patients with PHA than HCC were recorded (85.8% vs. 74.6%, *p* < 0.0001). Overall, patients with PHA had fewer comorbidities, with 66.2% of patients having a Charlson/Deyo score of 0, compared to 46.9% for patients diagnosed with HCC (*p* < 0.0001). The demographics of the patients diagnosed with HCC and PHA are detailed in Table 1. The median survival for patients with HCC was 10.3 months (95% CI: 10.2–10.5) and 1.9 months (1.8–2.4) for patients with PHA (Figure 1), and this correlated with a higher risk of death for patients with PHA compared to those with HCC (aHR 2.41 (95% CI) (2.1–2.77), *p* < 0.0001) (Table 4).

## 4. Discussion

### 4.1. Primary Hepatic Angiosarcoma

PHA is a rare disease whose pathogenesis, clinical characteristics, and treatment outcomes are described only in case reports and small case series [3,9,10,13]. Ours is the first database study exclusively addressing the demographics and treatment outcomes of patients diagnosed with PHA. The NCDB contains data from approximately 1500 participating centers, representing roughly 70% of all oncologic cases in the United States [14]. The incidence of PHAs amongst the primary hepatic tumors reported here is derived from the NCDB database (2004–2014). Individual cancer registries report a 0.04 to 2% incidence of PHAs amongst hepatic tumors [15,16]. We found the incidence of PHAs was 0.29% amongst all primary hepatic tumors. The majority of patients diagnosed with PHA identified themselves as white race (85.8%), and 11.3% of patients were of Hispanic race. Thus far, PHAs have been reported to exhibit a male preponderance (2–4:1 ratio favoring men) and occur between the fifth and seventh decades of life [10,11,15,17]. In our study, PHAs were diagnosed at a mean age of 62.9 years and showed a 2:1 male preponderance, which is congruent with the reported literature.

The median OS of patients with PHA in our study was 1.9 months. Retrospective studies and systematic reviews report a median OS of approximately six months in patients diagnosed with PHA [9,10,17]. Groeschl et al., in their analysis of the SEER database (1975–2007), reported an overall median OS of 1 month in the 207 patients with PHA [18]. Retrospective studies and case series report a median OS of 6 months for patients diagnosed with PHA. This discrepancy in results could be because individual case reports and case series are usually derived from academic centers where patients have access to tertiary care [9,11,12,19]. When treated at ‘high-volume centers’, patients diagnosed with sarcoma have a better outcome than those treated at non-academic centers [20,21]. Although our results show that the patients treated in academic centers had a numerically higher medical survival than those treated at non-academic centers, these results were not statistically significant. One probable reason for this result could be the difference in definitions of academic centers as defined in the NCDB vis-à-vis the definition used in other studies. Studies that had access to more granular data had strict criteria to define ‘high-volume centers.’ On the other hand, the NCDB dictionary defines an Academic Comprehensive Cancer Program (ACCP) as any facility that encounters more than 500 cancers per year and participates in post-graduate medical education in at least four subjects (hematology–oncology training is NOT a must). Sarcoma is a rare tumor, and it is hard to ascertain if all facilities falling under an ACCP, or even otherwise, have a multidisciplinary tumor board or not. Other authors have also reported such discrepancies in their cohorts, and it is a limitation of big-data analysis [22].

Our results show that patients who received surgery had improved survival compared to those who did not. These results are congruent with the general principles of managing patients with PHAs, where surgical resection affords the best survival outcomes [7,8,23,24,25]. The existing literature also reflects better survival outcomes for patients diagnosed with PHAs who undergo surgical resection with or without locoregional therapies, such as transarterial chemoembolization (TACE) [9,10,11,18,26]. In patients with lesser stage tumors (Stage 1b) who underwent surgery with or without TACE, the median survival can extend beyond five years [9,12,27,28,29]. Wilson et al. retrospectively reviewed 44 patients diagnosed with PHA. Six out of eight patients presenting with resectable disease underwent an R0 resection in this cohort. Only two patients who underwent an R0 resection were alive at the end of five years. Patients undergoing a surgical resection for PHA had a median OS of 33.4 months compared to a median OS of 9.3 months for those who underwent locoregional therapy and 7.7 months for those who received chemotherapy. The patients who did not receive any treatment had the worst median OS of 1.9 months [9]. In another cohort of 5 patients with solitary PHA who underwent surgical resection, the 1-, 3-, and 5-year survival was 100%, 80%, and 40%, respectively. Although four out of five patients had a tumor recurrence (death recorded between 23 to 69 months), the excellent 1-year survival validates the benefit of a surgical resection [12]. It is important to note that a surgical resection is usually offered to patients with localized disease in patients diagnosed with any soft-tissue sarcoma (STS) [7,30]. It is also known that patients with localized angiosarcoma have better survival than those with metastatic disease [7]. In the current study, it is challenging to ascertain if a possible early stage of presentation confounds the survival benefit seen with surgical resection or not.

Patients with PHA who received chemotherapy had better survival and a lower risk of death than those who did not receive any chemotherapy. Patients with metastatic disease and a locally advanced STS that are not amenable to curable surgery or RT are treated with cytotoxic chemotherapy [7,30]. However, the survival data in our study only indicate the initial benefit as the survival curves cross over by two years, indicating no long-term benefit. Many studies have shown an improvement in OS with chemotherapy in patients with angiosarcoma, especially in the metastatic stage [23,24,25]. Kim et al. retrospectively reviewed records of 11,415 patients with primary hepatic tumors, out of whom five were diagnosed with PHA. All five patients had metastatic disease at presentation in this cohort, and three died within three months of diagnosis. The other two patients, who were younger and had better performance status, received second and third lines of chemotherapy regimens and lived for 16 and 9 months, respectively [15]. Likewise, Wilson et al. also reported a better median OS in patients receiving chemotherapy than those who did not receive any treatment (7.7 months versus 1.9 months). A study by Huang et al. studied a cohort of 34 patients reported and noted a median OS of 41 months (IQR—20 months) among patients that received both surgery and chemotherapy (*n* = 7) compared to 3 months (IQR—9.35 months) for those who received chemotherapy only (*n* = 5) [11]. These results from individual cohorts of patients with PHA are consistent with our findings. More research is needed to understand the long-term outcomes of patients with systemic chemotherapy and if any additional benefit may arise from combining it with surgery.

### 4.2. Primary Hepatic Angiosarcoma versus Hepatocellular Carcinoma

HCC is the most common primary tumor of the liver and the fourth most common cause of tumor-related death worldwide [31]. The overall five-year survival for HCC is 18%, making it a lethal tumor [31]. On the other hand, PHA is reported to have only a 3% survival rate at the end of 2 years compared to cutaneous or extremity angiosarcoma [7]. Although both malignancies are associated with poor outcomes, no study has reported a head-to-head comparison of survival outcomes of patients with HCC and PHA. Ours is the first study to report a significantly worse survival for patients diagnosed with PHA than those diagnosed with HCC. Moreover, patients diagnosed with PHA are 2.23 times more likely to die from the diagnosis at any given point compared to those diagnosed with HCC. The data in this analysis extended to 2014, when significant advancements were being made in treating patients with HCC [32]. On the contrary, no significant advances came in the treatment of STS, except for the approval of pazopanib in 2014, which did not provide an OS benefit to patients diagnosed with any STS [33]. In the last five years, systemic options for angiosarcomas have increased with the inclusion of checkpoint inhibitors, and it would be interesting to see their impact on the survival of patients diagnosed with PHA.

A liver transplant is controversial in PHAs. Unlike HCC, where a liver transplant is an option for patients with limited tumor burden and satisfying the Milan Criteria [31], it is contraindicated in patients with PHA due to high rates of recurrence, infection, and high mortality. Orlando et al. reviewed 22 patients with PHA listed in the European Liver Transplant Registry who underwent a liver transplant [34]. The OS was reported at 7.2 ± 2.6 months, and all patients died by 23 months. The recurrence was recorded in 17 patients by 5.0 ± 2.6 months, all of whom were dead by 23 months. Five patients died from infection. In addition, multiple patients experienced transplant-related complications such as rejection (14 patients) and renal and respiratory failure (4 patients). A review of the published literature by the same authors also demonstrated the same dismal results. In a subset analysis of primary liver sarcomas recorded in the NCDB, the authors reported a trend toward better survival in patients with PHA who received a resection compared to those who received a liver transplant [35].

### 4.3. Limitations of the Study

This study has a few significant limitations. First, being a retrospective analysis, there is a potential to introduce selection bias. In addition to this, a large proportion of patients with missing records could potentially worsen this bias. Second, the NCDB dataset does not include many clinically relevant variables (such as performance status) and hence were not included in our model [14]. Third, we cannot account for miscoding or erroneous data that can be seen in large multi-institutional registries. In addition, it is challenging to ascertain the course of treatment beyond the first course of treatment from cancer registries, limiting our analysis to assess the impact of subsequent treatments on overall survival. Moreover, many treatment-related variables such as the extent of surgery or the use of RT did not meet our prespecified criteria and could not be included in the analysis.

## 5. Conclusions

PHA is an aggressive malignancy with a significantly worse prognosis compared to HCC. Surgery and chemotherapy are associated with better survival outcomes in patients with PHA; however, the long-term benefits of chemotherapy are not clear. More research is needed to help determine the optimal treatment approaches for this very rare disease.

## Figures and Tables

**Table 1:** Descriptive statistics for Primary Hepatic Angiosarcoma and Hepatocellular Carcinoma. National Cancer Database 2004–2014.

	Histologic Type		
	Angiosarcoma	HCC	*p*
Overall *n*	346	118,066	
Age, Mean (SD)	62.9 (13.7)	62.4 (11.1)	0.458
Sex, *n* (%)			
Male	224 (64.7%)	90,367 (76.8%)	<0.0001
Female	122 (35.3%)	27,266 (23.2%)	
Race, *n* (%)			
White	290 (85.8%)	85,200 (7.4%)	<0.0001
Black	25 (7.4%)	18,712 (15.9%)	
American Indian	1 (0.3%)	839 (0.7%)	
Asian and Pacific Islander	22 (6.5%)	9477 (8.1%)	
Ethnicity, *n* (%)			
Non Spanish	284 (88.8%)	97,216 (86.5%)	0.245
Hispanic	36 (11.3%)	15,142 (13.5%)	
Metro Status, *n* (%)			
Metro	270 (81.1%)	99,948 (87.3%)	0.002
Urban	54 (16.2%)	12,986 (11.5%)	
Rural	9 (2.7%)	1470 (1.3%)	
Insurance Status, *n* (%)			
Not Insured	15 (4.5%)	7772 (6.8%)	<0.0001
Private Insurance	146 (43.7%)	38,481 (33.5%)	
Medicaid	22 (6.6%)	17,587 (15.3%)	
Medicare	145 (43.4%)	48,763 (42.5%)	
Other Government	6 (1.8%)	2120 (1.9%)	
Region, *n* (%)			
Northeast	63 (19.1%)	24,451 (21.1%)	<0.0001
Midwest	104 (31.6%)	22,811 (19.7%)	
South	98 (29.8%)	45,337 (39.1%)	
West	64 (19.5%)	23,382 (20.2%)	
Facility Type, *n* (%)			
Community Cancer Program	21 (6.4%)	6335 (5.5%)	0.141
Comprehensive Community Cancer Program	104 (31.6%)	32,083 (27.7%)	
Academic/Research Program	164 (49.8%)	65,237 (56.3%)	
Integrated Network Cancer Program	40 (12.2%)	12,326 (10.6%)	
Charlson/Deyo Score			
0	229 (66.2%)	55,031 (46.8%)	<0.0001
1	57 (16.5%)	31,722 (27.0%)	
2	20 (5.8%)	12,403 (10.5%)	
≥3	40 (11.6%)	18,477 (15.7)	
Metastasis	43 (26.2%)	5407 (8.6%)	<0.0001
Bone	24 (13.0%)	2509 (3.8%)	
Lung	19 (10.3%)	2997 (4.5%)	
Treatment Modalities used			
Surgery, *n* (%)	50 (14.6%)	31,248 (26.7%)	<0.0001
Radiation, *n* (%)	12 (3.5%)	9946 (8.5%)	0.001
Chemotherapy, *n* (%)			
No	218 (64.3%)	56,023 (55.0%)	0.086
Yes (single agent)	82 (24.2%)	34,755 (34.1%)	
Yes (multi-agent)	39 (11.5%)	11,161 (11.0)	
Alive, *n* (%)	34 (9.8%)	31,158 (26.5%)	<0.0001

**Table 2:** Descriptive Statistics: National Cancer Database 2004–2014, Primary Hepatic Angiosarcoma stratified by Surgery and Chemotherapy Status.

	Received Surgery			Received Chemotherapy		
	No	Yes	*p*	No	Yes	*p*
**Overall *n***	293 (85.4%)	50 (14.6%)		218 (64.3%)	121 (42.0%)	
**Age, Mean (SD)**	64.2 (13.4)	55.9 (13.4)	<0.0001	65.0 (13.7)	59.7 (12.6)	<0.0001
**Sex, *n* (%)**						
**Male**	198 (67.6%)	25 (50%)	0.016	140 (64.2%)	79 (65.3%)	0.84
**Female**	95 (32.4%)	25 (40%)		78 (35.8%)	42 (34.7%)	
**Race, *n* (%)**						
**White**	245 (83.6%)	44 (88.0%)	0.93	184 (84.4%)	100 (82.6%)	0.85
**Black**	21 (7.2%)	3 (6.0%)		14 (6.4%)	11 (9.1%)	
**American Indian**	1 (0.3%)	0 (0%)		1 (0.5%)	0 (0%)	
**Asian and Pacific Islander**	20 (6.8%)	2 (4.0%)		14 (6.4%)	7 (5.8%)	
**Ethnicity, *n* (%)**						
**Non-Spanish**	243 (89.7%)	39 (83.0%)	0.18	175 (87.5%)	102 (90.3%)	0.46
**Hispanic**	28 (10.3%)	8 (17.0%)		25 (12.5%)	11 (9.7%)	
**Metro Status, *n* (%)**						
**Metro**	231 (81.9%)	36 (75.0%)	0.51	166 (79.1%)	98 (84.5%)	0.28
**Urban**	44 (15.6%)	9 (20.8%)		39 (18.6%)	14 (12.0%)	
**Rural**	7 (2.5%)	2 (4.2%)		5 (2.4%)	4 (3.5%)	
**Insurance Status, *n* (%)**						
**Not Insured**	11 (3.9%)	3 (6.1%)	0.099	10 (4.7%)	4 (3.5%)	0.004
**Private Insurance**	118 (41.8%)	27 (55.1%)		79 (36.7%)	64 (56.6%)	
**Medicaid**	18 (6.4%)	4 (8.3%)		20 (9.3%)	2 (1.8%)	
**Medicare**	131 (46.5%)	13 (26.5%)		102 (47.4%)	41 (36.8%)	
**Other Government**	4 (1.4%)	2 (4.1%)		4 (1.9%)	2 (1.8%)	
**Region, *n* (%)**						
**Northeast**	54 (19.3%)	7 (15.2%)	0.18	39 (18.5%)	24 (21.2%)	0.004
**Midwest**	89 (31.8%)	15 (32.6%)		55 (216.1%)	47 (41.6%)	
**South**	78 (27.9%)	19 (41.3%)		66 (31.3%)	30 (26.6%)	
**West**	59 (21.1%)	5 (10.9%)		51 (24.2%)	12 (106%)	
**Facility Type, *n* (%)**						
**Community Cancer Program**	19 (6.8%)	1 (2.2%)	<0.0001	17 (8.1%)	4 (3.5%)	0.097
**Comprehensive Community Cancer Program**	99 (35.4%)	4 (8.7%)		73 (34.6%)	29 (25.7%)	
**Academic/Research Program**	127 (45.4%)	36 (78.3%)		98 (46.5%)	65 (57.5%)	
**Integrated Network Cancer Program**	35 (12.5%)	5 (10.9%)		23 (10.9%)	15 (13.3%)	
**Charlson/Deyo Score**						
**0**	192 (65.5%)	34 (68.0%)	0.83	131 (60.1%)	94 (77.7%)	0.002
**1**	50 (17.1%)	7 (14.0%)		36 (16.5%)	18 (14.9%)	
**2**	16 (5.5%)	4 (8.0%)		17 (7.8%)	6 (5.0%)	
**≥3**	35 (12.0%)	5 (10%)		34 (15.6%)	33 (12.5%)	
**Metastasis**	42 (29.0%)	1 (5.3%)	0.027	16 (22.3%)	21 (37.5%)	0.073
**Radiation, *n* (%)**	10 (3.4%)	2 (4.0%)	0.84	5 (2.3%)	6 (5.0%)	0.39
**Chemotherapy, *n* (%)**	109 (45.6%)	12 (26.1%)	0.014	--	--	
**Surgery, *n* (%)**	--		--	34 (20.7%)	12 (9.9%)	0.014
**Alive, *n* (%)**	20 (6.8%)	13 (26.0%)	<0.001	23 (10.6%)	11 (9.1%)	0.44

**Table 3:** Median Survival and Cox Proportional Hazards Models for surgery and chemotherapy status for Primary Angiosarcoma of Liver. National Cancer Database 2004–2014.

	Median Survival	Cox Proportional Hazards			
	Median Survival (95% CI)	Univariate HR (95% CI)	*p*	Multivariable HR *,† (95% CI)	*p*
**Surgery Status**					
**Yes**	7.69 (5.46–16.92)	0.34 (0.24–0.49)	<0.0001	0.23 (0.15–0.37)	<0.0001
**No**	1.77 (1.48–1.94)	Ref		Ref	
**Chemotherapy Status**					
**Yes**	5.09 (3.77–5.81)	0.59 (0.47–0.76)	0.001	0.44 (0.32–0.60)	<0.0001
**No**	1.15 (0.85–1.61)	Ref		Ref	

* Surgery Status—Adjusted for Age, Race, Ethnicity, Sex, Urbanicity, Facility Location and Type, Charlson/Deyo Score, Chemotherapy, and Radiation. † Chemotherapy Status—Adjusted for Age, Race, Ethnicity, Sex, Urbanicity, Facility Location and Type, Charlson/Deyo Score, Surgery, and Radiation.

**Table 4:** Median Survival and Cox Proportional Hazards Models for Primary Angiosarcoma and HCC of Liver. National Cancer Database 2004–2014.

	Median Survival (Months)	Cox Proportional Hazards			
	Median Survival (95% CI)	Univariate HR (95% CI)	*p*	Multivariable HR * (95% CI)	*p*
**Cancer Type**					
**Angiosarcoma**	1.94 (1.77–2.36)	2.39 (2.13–2.67)	<0.0001	2.41 (2.10–2.77)	<0.0001
**HCC**	10.35 (10.22–10.51)	Ref		Ref	

* Surgery Status—Adjusted for Age, Race, Ethnicity, Sex, Urbanicity, Facility Location and Type, Charlson/Deyo Score, Chemotherapy, and Radiation.

## Data Availability

Not applicable.
